# Complications of oxidised regenerated cellulose at Caesarean section: A report of two cases

**DOI:** 10.52054/FVVO.14.4.050

**Published:** 2023-01-27

**Authors:** A Ward, P Ives, A Koumousidis

**Affiliations:** Obstetrics and Gynaecology Department, Women and Children’s Division, Conquest Hospital, The Ridge, St Leaonard’s, TN37 7RD, East Sussex NHS Health Care NHS Trust

## Abstract

Two patients underwent surgical deliveries within four months of one another at a single maternity unit. Both patients had complications of infection-like symptoms such as offensive vaginal discharge and pyrexia, months following their caesarean sections resulting in further surgery. The incidents were thought to be secondary to woven oxidised regenerated cellulose (ORC) use. ORC must be used according to its relevant product literature which can vary between brands. Surgeons must keep abreast of changes to the haemostatic material provided to them and therefore the properties of each type, especially when faced with bleeding not suitable for suturing or electrocautery.

## Introduction

Oxidised cellulose first came into clinical use in 1942 (Frantz, 1945) and exerts its haemostatic effects by forming a physical matrix to enhance clot formation (Schreiber and Neveleff, 2011). The material’s low pH enhances the extrinsic arm of the coagulation pathway while simultaneously achieving bactericidal properties (Achneck et al., 2010). Often manufactured from lyocell, a fibrous reconstituted cellulose derivative of wood pulp (Kollar et al., 2008), oxidised regenerated cellulose (ORC) has been used safely in a range of surgical specialties for its ability to stop venous oozing where other methods are inappropriate (Hutchinson et al., 2013) e.g., suturing or electrocautery close to nerves. Complications reported in the literature include delayed paraplegia when used intra- spinally (Brodbelt et al., 2002) and migration in tracheoesophageal fistula repair (Dokumcu et al., 2014), however, there are few case reports of complications of ORC at caesarean section. We present two patients who required surgical intervention at six and eight months post-surgical delivery respectively for ongoing symptoms of vaginal discharge and febrile illness. Possible causes are discussed, including examination of the manufacturers’ instructions for use. The authors hope to increase awareness of this safety issue with a literature review and a review of up-to-date guidelines. Written consent was obtained from the patients to write up and research their cases.

## Case Presentation

### Case 1

A 26-year-old woman with a body mass index (BMI) of 28 underwent an emergency caesarean section for foetal distress. She was otherwise fit and well, was taking no regular medications and had no previous surgery to her abdomen. The delivery at caesarean was uncomplicated and the uterine incision was closed in two layers. Estimated blood loss was 450mls but the lower uterine segment and pre-vesical fat beneath the peritoneal bladder reflection were persistently oozing despite multiple superficial haemostatic sutures and a well contracted uterus. There was no suspicion of a bladder injury and therefore methylene blue testing or cystoscopy was not performed. A haemostatic material, oxidised regenerated cellulose (ORC) was inserted at the base of the bladder reflection and to the left uterine angle. Haemostasis was achieved. The remaining closure of the abdomen was unremarkable.

Seven weeks following the birth of her baby, the patient presented with painless persistent vaginal bleeding. The patient was exclusively feeding her infant with formula and had not yet returned to a normal menstrual cycle. She had no lower urinary tract symptoms. Observations were normal and her urine pregnancy test was negative. Blood tests revealed a White Cell Count (WCC) of 7.94 x109/L and a C-Reactive Protein (CRP) of 3mg/L. High vaginal swab (HVS) revealed light growth of gut flora. A trans-vaginal ultrasound (TVUS) revealed a 31x20x28mm ‘large mass of suture material at the caesarean section site incision (uterine scar)’. She was discharged with a course of oral antibiotics.

Six weeks following her initial consultation, the patient presented with an offensive vaginal discharge and passage of what appeared to be suture material, thought to have been the oxidised regenerated cellulose material used during her caesarean. She was haemodynamically stable and afebrile. An incident form was completed. Her blood tests showed no change from those six weeks earlier. She was discharged with a seven- day course of oral cephalexin and metronidazole, however, re-presented with more foul-smelling discharge and a history of fever three weeks later. On this occasion her WCC had increased to 15.79 x109/L. A high vaginal swab showed a coincidental picture of bacterial vaginosis. She was given another 5-day course of antibiotics and underwent an outpatient TVUS. The report described a 24x13x24mm ‘heterogenous well defined mass’ and so was referred to the benign gynaecological multi-disciplinary team (MDT) who requested a magnetic resonance imaging (MRI) study of the pelvis as an outpatient.magnetic resonance imaging (MRI) study of the pelvis as an outpatient.

**Figure 1:**
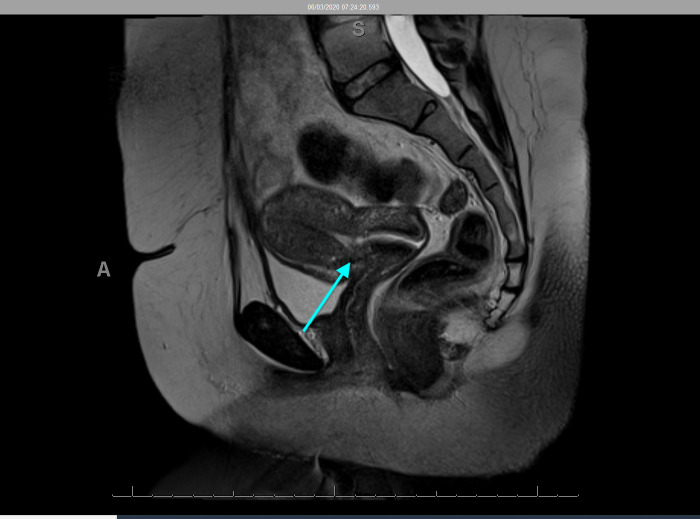
Case 1 Sagittal section of MRI demonstrating a possible niche and or fistula between the uterus and peritoneal cavity.

The MRI of her pelvis revealed a possible niche and/or fistula between the uterus and the peritoneal cavity via the caesarean scar, without bladder involvement (Figure 1). Considering her persistent symptoms, the MDT recommended a hysteroscopy and laparoscopy to investigate further and treat any underlying pathology such as incision and drainage of abscess or closure of a uterine fistula.

Due to the coronavirus pandemic, her surgery was significantly delayed up to 6 months after her initial presentation. The intra-operative findings of hysteroscopy revealed a niche in the right anterior portion of the cervico-uterine junction. At laparoscopy, a utero-peritoneal (metroperitoneal) fistula was identified while using the hysteroscope and laparoscope simultaneously. No foreign body or haemostatic material was found. Overlying the fistula were dense adhesions involving the omentum and bladder. The omentum was dissected down, however it was felt that it was not safe to proceed with dissecting the uterus off the bladder due to the risk of inadvertent bladder perforation. Therefore, the patient was treated conservatively with a prolonged course of antibiotics and referred for a repeat MRI scan in 6 months. A secondary plan was put in place for a referral to a gynaecologist with an interest in hysteroscopic surgery, had the niche/fistula not improved. The patient was also advised to use contraception for at least one year to prevent caesarean scar dehiscence and symptoms of uterine niches, such as intermenstrual bleeding. The repeat MRI 6 months later showed the uterine myometrium at the area of the scar had thickened to 5mm. At the time of writing, the patient was 24 weeks into her second pregnancy.

### Case 2

A 35-year-old woman with a BMI of 25 with a history of one previous caesarean section underwent a planned caesarean section at 39 weeks gestation. Intra-operatively, the bladder was noted to be high on the lower segment but was dissected down without complications. The estimated blood loss was 350mls. ORC was used for a persistently oozing lower uterine segment.

**Figure 2:**
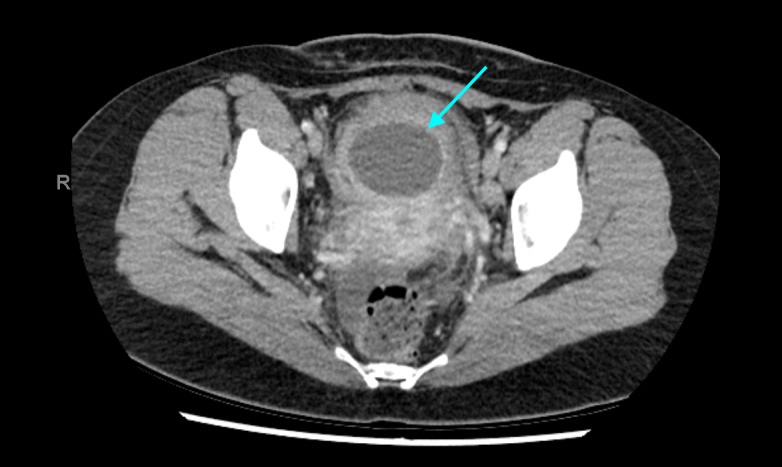
Case 2 Transverse section of CT demonstrating a thick-walled collection between the uterus and the bladder.

**Figure 3:**
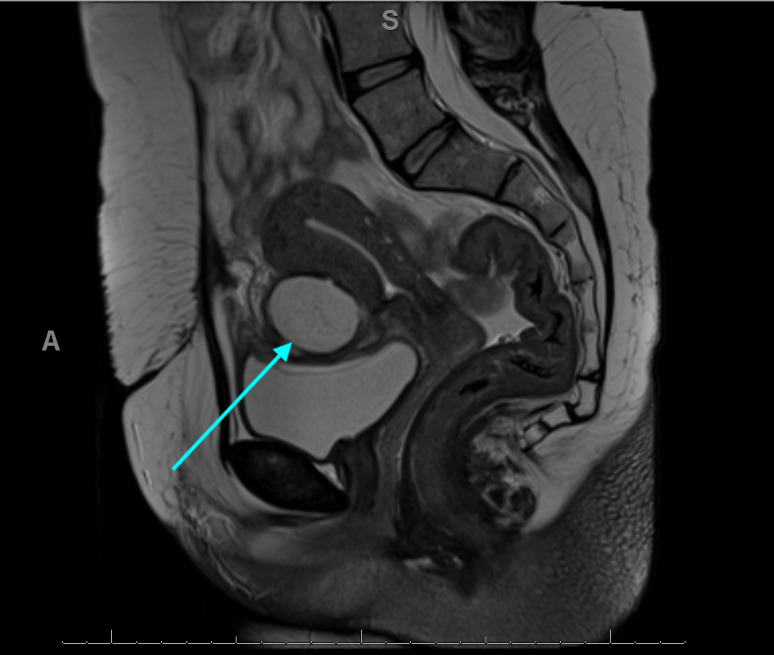
Case 2 Sagittal section of MRI demonstrating a fluid collection at the uterine vesicular fold closely related to the caesarean section scar.

Four weeks later, the patient presented to Accident and Emergency Department with lower abdominal pain. A computed tomography (CT) scan of her abdomen and pelvis revealed a 66x53x19mm collection in the uterovesicular (UV) fold (Figure 2). She was treated with intravenous antibiotics and once she was improving, was discharged with one week of oral antibiotics. Four months later, the patient re-presented with lower abdominal pain and dyspareunia. A CT scan revealed a 45mm thick- walled collection thought to be an abscess between the uterus and the bladder. As the collection had shown a reduction in size and there were no signs of overt sepsis, the patient was discharged with a course of antibiotics for six weeks and a plan to undergo a subsequent repeat MRI. Choice of antibiotics was based on local antimicrobial policy, rather than culture as achieving ultrasound guided or CT guided aspiration of the collection was deemed too high risk due to its relationship with overlying bladder and bowel. The MRI revealed a fluid collection at the UV fold, closely applied to the caesarean section scar (Figure 3). The radiologist described the collection as not characteristic of a foreign body or suture material but commented that it was slowly decreasing in size. A uterine niche or fistula did not make up part of the radiological differential diagnosis. At telephone clinic, the patient was asymptomatic, so was discharged to the care of her general practitioner.

Two months following her telephone consultation, eight months on from her caesarean section, the patient represented for the third time with lower abdominal pain and signs of infection. Blood tests revealed a WCC of 17.9 x109/L and a CRP of 434mg/L. A multidisciplinary decision was made to perform a laparoscopy to drain the potential collection. Intra-operatively, the bladder was adherent to the uterus. The bladder was carefully dissected down and in between the UV fold and bladder was found a jelly like material, which on close inspection was made of fibrous like material. Histology revealed a scanty acellular substance.

## Discussion

In this article we present two patients who presented with similar complaints of pain, vaginal discharge and infection-like symptoms following caesarean delivery. They occurred within four months of one another. One patient presented with passage of a possible foreign body despite that instruments, sharps and swab counts were correct at the end of each procedure. Due to the similarities of the cases, a route cause analysis was performed. Prior to the investigation, it was thought by members of the obstetric and gynaecological surgical team that the cause of these complications was due to a change in the type of the haemostatic material used in the obstetric theatre procured by the hospital’s surgical services unit. On systematic analysis by the incident review committee, this particular type of ORC had replaced the original brand of haemostatic material, well known to the obstetric and gynaecological surgeons in 2014, five years prior to these caesareans. The change in the haemostatic material however was not known to any of the operating surgeons, who may have changed their practice had they known or read the relevant product literature to ensure they used it safely. However, other specimens of the ORC with the same lot number had also been used within different surgical specialities in the trust without complications. Both caesareans were performed by different surgeons with different theatre scrub nurses. Unfortunately, the exact way in which the ORC was applied and used intra-operatively is unknown as there was minimal description in the operative notes and the procedures were not recorded visually. However, it is possible that a sheet of 3x4 inch woven material would be packed and pressed into the area and left in situ. We propose that ‘overpacking’ resulted in expansion of the material within a confined space. Although ORC has antibacterial properties, if infection was also introduced simultaneously with excess blood and clots acting as a nutrient base for bacteria, this may have significantly impaired the absorption of the material. It is thought that in the case in which the patient passed the haemostatic material vaginally, that the ORC may have travelled through the uterine scar with uterine involution and then, passed through the cervix.

The product literature of the haemostatic material discussed above states that it is suitable for all types of surgery. It also outlines that “The gauze may remain in situ and the wound may be closed since the gauze completely decomposes within one week” (Equimedical, 2008). The literature informs the reader, that the product should be absorbed within a maximum of 30 days and normally within 8 days. In our cases, both patients presented with symptoms once 30 days had elapsed from their surgery. With regards to infection, “Equitamp (Equimedical, 2008) may form a nidus of infection, it must not be left in infected areas; it must be removed once the bleeding has been controlled”. One case was an elective procedure, the other was an emergency however, there were no signs of infection noted intra-operatively at either procedure. The product information leaflet also describes; “Care should be exercised to avoid overpacking” as the gauze expands on absorption of liquid (Equimedical, 2008). Interestingly, Surgicel (Ethicon, 2022), uses the same phrase for its indication as Equitamp; “To assist in the control of capillary, venous, small arterial haemorrhage when ligation or other conventional methods of control are impractical or ineffective” (Ethicon, 2022). However, this product differs, in that, although it can be left in situ where necessary, the manufacturer advises removal of the material once haemostasis is achieved. It also recommends removing any excess material before surgical closure, to minimise the possibility of foreign body reaction (Ethicon, 2022).

Oxidised regenerated cellulose (ORC) was developed in 1960 and has been used extensively in neurosurgery due its ability to stop continuous venous bleeding (Schonauer et al., 2004). Almost all forms of commercially available ORC have studies and case reports to support their use in their relevant specialities (Sharma and Malhotra, 2006), however as with all types of surgical device, complications do occur. Indeed, both product literatures described above advise against leaving ORC in or around bony foramina and the spinal cord, due to swelling of the material exerting pressure on surrounding structures (Equimedical, 2008; Ethicon, 2022). Menovsky et al. (2011) describes massive swelling of even small amounts of ORC compressing the dural space and migration of ORC has also been reported through intervertebral foramen (Kanakis et al., 2013). As a result of safety concerns and an ever-growing range of haemostatic materials available, Osvaldo et al. (2018) provides a systematic review on topical haemostats, their properties, indications, and contra-indications.

In gynaecological oncology, Fagotti et al. (2010) found a relationship between pelvic exenterations, ORC use and pelvic abscesses including one case presenting with a persistent collection of ORC 15 months post-surgery. Within obstetrics, Abraham (2017) reported very similar outcomes in a retrospective observational study of women who underwent caesarean with ORC and women who underwent caesarean without ORC, however, there was an increased incidence of post operative pyrexia in the ORC group. Only one similar case report was found in the literature review by Scaffidi and McMicking (2018) who describes a patient passing ORC vaginally 8 days post emergency caesarean section for suspected chorio-amnionitis. The American College of Obstetricians and Gynaecologists released a committee opinion paper in October 2020 which acknowledged the lack of data around the subject of topical haemostatic materials in obstetrics and gynaecology. It highlighted that haemostatic agents should not be a substitute for meticulous surgical technique and should only be used in locations where stitches or electrocautery may not be safe e.g., adjacent to nerves. It also describes the importance of understanding the different agents’ mechanisms of actions and their relative cost benefit analyses (ACOG, 2020).

Locally, as a result of the investigation, oxidised regenerated cellulose materials of any brand were removed from the obstetric theatre and replaced with a popular biological haemostatic agent composed of gelatine granules and thrombin. These are thought to be of greater use in patients with coagulation difficulties and/or in those where bleeding is more active (Hanks et al., 2003). They are of greater cost however and some types that contain human or animal thrombin may not be accepted by those who decline blood products or those of certain religious beliefs (RCSE, 2016). The results of the investigation and subsequent intervention were communicated to the patients involved as part of a duty of candour process.

## Conclusion

Inappropriate use of ORC may cause infectious complications following caesarean sections, resulting in prolonged symptoms. Clinicians need to be aware of these complications and familiarise themselves with the correct use of this haemostatic agent.

Surgeons must keep abreast of changes to the haemostatic materials that will be offered to them intra-operatively. They must be familiar with the product’s literature as each type and subtype will behave differently. This is key for effective haemostasis and surgical safety.
